# Amorphous Poly (Aryl Ether Ketones) Containing Methylene Groups with Excellent Thermal Resistance, Dielectric Properties and Mechanical Performance

**DOI:** 10.3390/polym15214330

**Published:** 2023-11-06

**Authors:** Jingwei Jiang, Zhichao Wang, Yunlong Sun, Zengxu Qian, Zengwen Cao, Zhipeng Wang, Guangyuan Zhou

**Affiliations:** 1 Division of Energy Materials (DNL22), Dalian Institute of Chemical Physics of the Chinese Academy of Sciences, Dalian 116023, China; jwjiang@dicp.ac.cn (J.J.); sunyl@dicp.ac.cn (Y.S.); qianzx@dicp.ac.cn (Z.Q.); zwcao@dicp.ac.cn (Z.C.); 2 School of Textile and Material Engineering, Dalian Polytechnic University, Dalian 116034, China; wangzc@dlpu.edu.cn

**Keywords:** poly (aryl ether ketone), methylene groups, dielectric constant, dielectric loss

## Abstract

Low-dielectric constant polymers are widely used in various microelectronic materials. With the development of 5G communication technology, there is an urgent need for polymer materials with low dielectric constant at high frequency, good thermal resistance, and mechanical properties. In this study, four novel poly (aryl ether ketone) (PAEK) containing different numbers of methylene groups were synthesized via nucleophilic polycondensation reaction. At 10 GHz, these polymer films exhibit excellent dielectric properties with dielectric constants as low as 2.76. The relationship between the dielectric constant and the number of methylene groups is illustrated by constructing the amorphous accumulation cell model. In addition, methylene groups provided the polymer with favorable mechanical performance, including Young’s modulus in the range of 2.17–2.21 GPa, the tensile strength from 82.0 to 88.5 MPa and the elongation at the break achieved 7.94%, respectively. Simultaneously, the polymer maintains good thermal resistance with a glass transition temperature (*T_g_*) reaching 216 °C. The result indicates that the obtained novel PAEK is potentially valuable in the field of high-frequency communications.

## 1. Introduction

The 5th Generation Mobile Communication Technology (5G) is a new generation of broadband mobile communication technology with high speed, low latency, and large connectivity points. High-frequency transmission technology is one of the key technologies of 5G communication, which can increase the utilization rate of frequency band resources and enhance the technical demand of 5G wireless communication technology for network development [1,2,3]. Additionally, high-frequency transmission requires the use of materials with a low dielectric constant and dielectric loss [4,5,6,7]. Therefore, there is an increasing demand for materials with low dielectric constant and dielectric loss at high frequencies.

The traditional commercially available low-dielectric materials include polytetrafluoroethylene [8,9], polyethylene [10,11], and polypropylene [12,13]. They have low dielectric constant and dielectric loss, but their thermal stability still needs to be enhanced. Thus, the design of polymers with excellent thermal stability, flexibility, low dielectric constants, and low dielectric losses became a key challenge. Poly (aryl ether ketone) (PAEK) is a thermoplastic resin with excellent performance [14,15,16,17]. Due to its unique rigid structural characteristics, it has excellent thermal stability, dimensional stability, and mechanical properties and can be widely used in aviation, aerospace, and electronic information fields [17,18,19,20]. However, the conventional PAEK shows high dielectric constant and dielectric loss. In recent years, researchers have reduced the dielectric constant and dielectric loss of PAEK using modification methods [21]. For example, PAEK with low dielectric constant and dielectric loss were prepared by introducing porous materials [5,22]. Nevertheless, problems such as the size and distribution of the porous fillers lead to their poor reproducibility, and the introduction of the porous structure weakens the mechanical properties of the material [23,24,25]. Consequently, it became more important to design and prepare intrinsic polymers with low dielectric constant and dielectric loss.

There are two means of lowering the dielectric constant of polymers that are based on molecular structure design. One is to introduce substituent groups with low polarizability, such as the F-substituent and methylene groups, which can decrease the polarizability of the polymer and thereby lower its dielectric constant [21]. Liu et al. synthesized semi-aromatic polyamides containing different numbers of methylene groups. The result found that the dielectric constant of the polyamides is effectively reduced by introducing low polarizability methylene groups [26]. Another strategy is to introduce non-polar rigid prudent groups, such as fluorene groups, phenyl groups, adamantane groups, and phenolphthalein groups [21]. The introduction of non-polar rigid prudent groups decreases the stacking density of the molecular chain and increases the free volume of the polymer, thus reducing the dielectric constant of the polymer [27]. Zhu et al. designed and prepared a series of PAEK-containing fluorene groups. The rigid twisted prudent fluorene group provides the polymer with a large free volume. As a result, the polymers exhibit excellent dielectric properties with a dielectric constant in the range from 2.41 to 3.52 and dielectric loss in the range from 0.005 to 0.029 at 1 GHz to 10 GHz [28]. Ling et al. introduced bulky isopropyl and fluorene groups into PAEK to increase the free volume. These polymer films have low dielectric constants (2.4–2.7) at 10 GHz to 42 GHz while maintaining excellent thermal resistance [1]. From the above studies, it was found that the introduction of non-polar rigid prudent groups and methylene groups into the polymer structure can effectively reduce the dielectric constant of the polymer. Thus, it is reasonable to consider that introducing low polarizability methylene groups and large free volume prudent groups can decrease the dielectric constant of PAEK. The rigid prudent groups maintain excellent thermal resistance of the polymer, and the flexible methylene group furnishes the polymer with favorable mechanical properties. Further, few studies have focused on the introduction of low polarizability methylene groups to obtain the low dielectric constant PAEK.

Conventional PAEK has excellent thermal resistance and mechanical properties. However, they exhibit high dielectric constant and dielectric loss at high frequencies, which is the main reason for limiting their use in the field of high-frequency communications. In order to prepare PAEK with excellent thermal resistance, mechanical and dielectric properties, this study designs and prepares difluoro-monomers 1,4-bis(4-fluorophenyl)butane-1,4-dione (BFB) and di-*p*-fluorophenyl-1,5 pentanedione-1,5 (DPFP) containing low polarizability methylene groups to reduce dielectric constant. A series of PAEK-containing methylene groups were synthesized using the nucleophilic polycondensation reaction of BFB, DPFP, 2-Phenyl-3,3-bis(4-hydroxyphenyl)phthalimidine (PPPBP), and 9,9-Bis(4-hydroxyphenyl)fluorene (BPF). The properties of PAEK were modulated by controlling the number of methylene groups in the difluoro-monomers. The effect of the number of methylene groups on the properties of PAEK was systematically investigated. Particularly, the relationship between the dielectric constant and methylene group amount was described by molecular dynamics simulation.

## 2. Experiment

### 2.1. Materials

Glutaronitrile (GTN, 98%) was purchased from Shanghai Macklin Biochemical Co., Ltd., Shanghai, China. Potassium 4-fluorophenyltrifluoroborate (FPFB, 97%), 2,2’-bipyridine (BPD, 97%), palladium(II) trifluoroacetate [Pd(CF_3_CO_2_)_2_, 98%], 2-bromo-1-(4-fluorophenyl)ethan-1-one (BFEO, 98%) and sodium hydroxymethanesulfinate dihydrate (SHMD, 98%) were purchased from Shanghai Bide Pharmatech Co., Ltd., Shanghai, China. 2-Phenyl-3,3-bis(4-hydroxyphenyl)phthalimidine (PPPBP, 99%) was obtained from Shanxi Liuqing Pharmaceutical Co., Ltd., Taiyuan, China. 9,9-Bis(4-hydroxyphenyl)fluorene (BPF, 98%), anhydrous potassium carbonate (K_2_CO_3_, 99%), and N,N-dimethylformamide (DMF, 99%) were purchased from Beijing Innochem Technology Co., Ltd., Beijing, China. Sulfolane (TMS, 99%) was purchased from Shanghai Aladdin Co., Ltd., Shanghai, China. Trifluoroacetic acid (TFA, 98%) was purchased from Anhui Energy Chemical Co., Ltd., Huangshan, China. Tetrahydrofuran (THF, 99%), N,N-dimethylacetamide (DMAc, 99%), Ethyl acetate (EA, 99%), and dichloromethane (DCM, 99%) were purchased from Guangzhou Guanghua Chemical Co., Ltd., Guangzhou, China. Toluene (99%), N-methylpyrrolidone (NMP, 98%), and Trichloromethane (CHCl_3_, 99%) were purchased from Tianjin Damao Chemical Co., Tianjin, China. Petroleum ether (PE, 99%) was purchased from Tianjin Fuyu Fine Chemical Co., Ltd., Tianjin, China. Unless otherwise specified, the materials used in the study, such as ethanol, were commercially available and used without further purification.

### 2.2. Synthesis of Monomer

#### 2.2.1. Synthesis of BFB Monomer

**Scheme 1:**
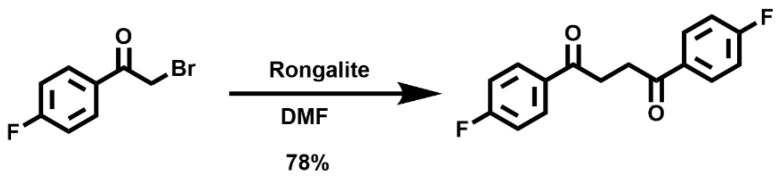
The synthesis route of BFB.

The synthesis of BFB was according to the literature (Scheme 1) [29]. A mixture of BFEO (26 g, 116 mol), SHMD (20 g, 123 mmol), and DMF (160 mL) at room temperature was stirred for 3 h. Deionized water (260 mL) was added, and the white solid was separated. The mixture was filtered through a glass sintered funnel and washed with deionized water (4 × 60 mL). The solid was dissolved in DCM, the solvent was removed again using a rotary evaporator, and the residue was purified by column chromatography on silica gel using DCM/PE as eluent (from 1:1 to 3:1 *v*/*v*) to obtain 24.8 g BFB product (white solid, yield: 78%). The characterization of BFB was shown in the Supporting information (Figures S1 and S2). ^1^H NMR (700 MHz, chloroform-*d*): δ 8.07 (dd, *J* = 8.4, 5.6 Hz, 4H), 7.15 (t, *J* = 8.4 Hz, 4H), δ 3.43 (s, 4H). The molecular weight for C_16_H_12_F_2_O_2_ [M]^+^: 274.0884, found: 274.0880 by high-resolution gas chromatography–mass spectroscopy.

#### 2.2.2. Synthesis of DPFP Monomer

**Scheme 2:**
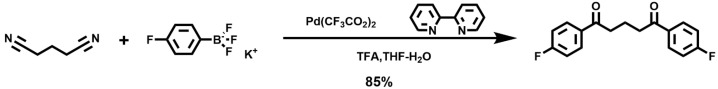
The synthesis route of DPFP.

The synthetic route of the DPFP monomer is shown in Scheme 2. Under N_2_ atmosphere, GTN (40 mmol, 3.78 mL), FPFB (160 mmol, 32.32 g), Pd(CF_3_CO_2_)_2_ (4.0 mmol, 1.33 mL), BPD (8.0 mmol, 1.25 g), TFA (46 mL), THF (200 mL) and H_2_O (40 mL) were added into a three-necked round bottom flask. The reaction mixture was stirred at 80 °C for 36 h. At the end of the reaction, the mixture was poured into ethyl acetate, which was neutralized with saturated NaHCO_3_. After the aqueous layer was extracted with ethyl acetate, dried with anhydrous Na_2_SO_4_, and the solvent was removed again using a rotary evaporator. The residue was purified by column chromatography on silica gel using PE/EA (7:1 *v*/*v*) as eluent to obtain 9.80 g DPFP product (white solid, yield: 85%). The characterization of DPFP was shown in the Supporting information (Figures S3 and S4). ^1^H NMR (700 MHz, chloroform-*d*): δ 8.01 (dd, *J* = 8.5, 5.6 Hz, 4H), 7.13 (t, *J* = 8.5 Hz, 4H), 3.09 (t, *J* = 6.9 Hz, 4H), 2.21–2.17 (m, *J* = 6.9 Hz, 2H). The molecular weight for C_17_H_14_F_2_O_2_ [M]^+^: 288.0962, found: 288.0955 by high-resolution gas chromatography–mass spectroscopy.

### 2.3. Synthesis of Polymers

**Scheme 3:**
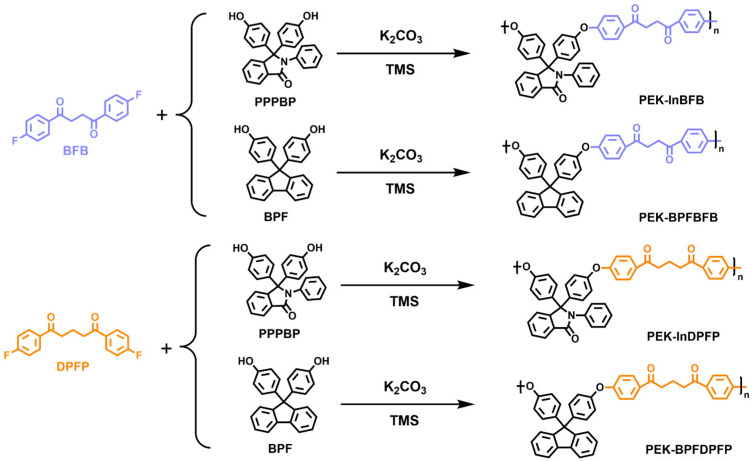
The synthetic routes of PEK-InBFB, PEK-BPFBFB, PEK-InDPFP, and PEK-BPFBFB.

All polymers were synthesized by a similar method (Scheme 3). The polymers prepared herein are named PEK-Inx and PEK-BPFx, where x is an abbreviation for difluoro-monomer. The synthesized PEK-InBFB is described as an example. Under N_2_ atmosphere, PPPBP (25 mmol, 9.8355 g), BFB (25 mmol, 6.8567 g), K_2_CO_3_ (28.75 mmol, 3.9734 g), TMS (37.33 mL), and 45 mL of toluene were added to a 250 mL three-necked round bottom flask with mechanical stirring and Dean-Stark trap fitted. The temperature of the reaction system was raised to 130 °C to form an azeotrope to remove the water from the flask. The temperature was increased to 135 °C for 1 h to eliminate the toluene in the system. After that, the temperature of the reaction system was elevated to 185 °C and kept for 2 h. The reaction is stopped by observing that the reaction solution becomes viscous. The reaction mixture was poured into a mixture of ethanol and water to obtain the precipitate. The precipitate was washed with boiling water and then dried in a vacuum oven at 170 °C for 24 h to obtain the polymers.

### 2.4. Preparation of Films

**Figure 1:**
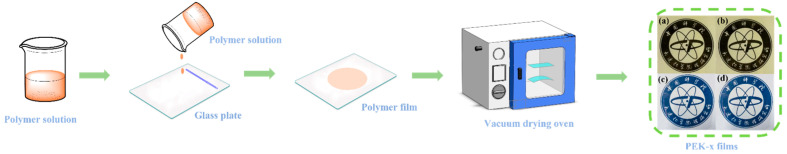
The preparation process of film samples. (**a**) PEK-InBFB film, (**b**) PEK-BPFBFB film, (**c**) PEK-InDPFP film, and (**d**) PEK-BPFDPFP film.

The PEK-Inx and PEK-BPFx films are prepared by the solution casting method (Figure 1). PEK-Inx and PEK-BPFx were dissolved in DMAc with a solid content of 20–25%. The mixture was dissolved for 12 h at room temperature. The polymer solution was filtered through a filter cloth and then cast in a clean glass plate. The glass plates were dried in an oven at 60 °C, 80 °C, 100 °C and 120 °C for 2 h, respectively. The film was peeled from a glass plate and dried in a vacuum oven at 180 °C for 24 h to obtain transparent films with a thickness from 50 to 70 µm.

### 2.5. Characterization

^1^H Nuclear magnetic resonance (NMR) spectra were recorded on a Bruker AVANCE III 700-MHz spectrometer (Bruker, Germany) with deuterated chloroform as the solvent. Fourier-transform infrared (FT-IR) spectra were recorded on a Nicolet Is50 spectrophotometer (Thermo Fisher Scientific, New York, NY, USA). The relative molecular weight of the polymer was determined on a Waters 2414 HPLC gel permeation chromatography (GPC) apparatus (Milford, MA, USA) and calibrated versus polystyrene standard by using THF as the eluent at a flow rate of 1.0 mL/min at 35 °C. The intrinsic viscosity [*η*] was measured using Ubbelohde viscometer with a 0.5 g/dL polymer solution in DMF at 25 °C. Wide-angle X-ray diffraction (WAXD) patterns of the films were recorded using a diffractometer (PANAlytical X’pert Pro-1, Almelo, The Netherlands) with an irradiation angle of 5–35°. Thermogravimetric analysis (TGA) was performed with a Discovery TGA 55 apparatus (TA, New Castle, DE, USA) under a nitrogen atmosphere at a heating rate of 10 °C min^−1^ from 30 to 700 °C. The glass transition temperature (*T_g_*) was obtained with differential scanning calorimetry (DSC 25, New Castle, DE, USA) under a nitrogen atmosphere at a heating rate of 10 °C min^−1^ from 30 to 300 °C. The tensile properties of the films were evaluated with a universal test machine (INSTRON 3366, Norwood, MA, USA) with 50 mm × 10 mm × 0.06 mm (length × width × thickness) film samples at a 2 mm/min speed. The dielectric properties of the films were measured with an Agilent E8362C PNA Microblog network analyzer at frequencies of 10 GHz. The films were pre-treated with ethanol to remove surface dust and then tested at (23 °C ± 2 °C) temperature and (50 ± 5%) humidity. The size of the sample films is 50 mm × 50 mm × 0.06 mm (length × width × thickness).

## 3. Results and Discussion

### 3.1. Structural Characterization

**Figure 2:**
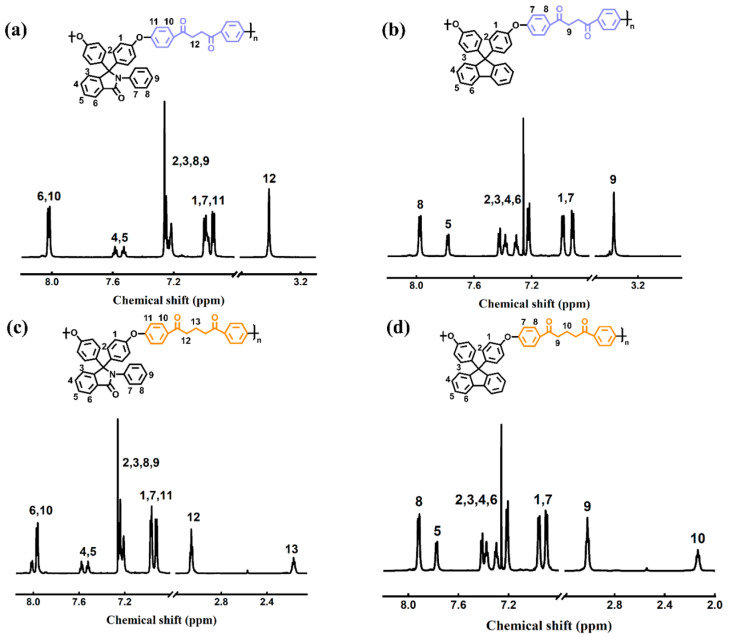
^1^H-NMR spectra of (**a**) PEK-InBFB, (**b**) PEK-BPFBFB, (**c**) PEK-InDPFP, and (**d**) PEK-BPFDPFP.

**Figure 3:**
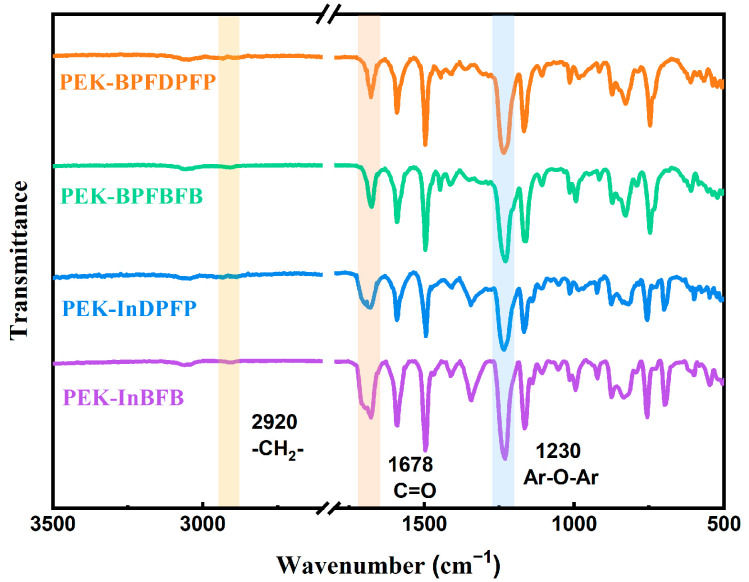
FT-IR spectra of PEK-Inx and PEK-BPFx.

**Figure 4:**
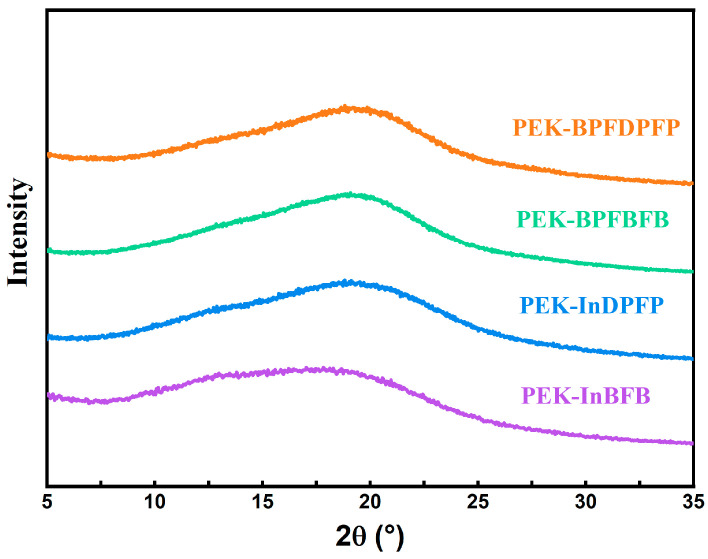
WAXD spectra of PEK-Inx and PEK-BPFx.

The chemical structures of PEK-Inx and PEK-BPFx were characterized by ^1^H NMR, FT-IR, and WAXD. The ^1^H NMR spectra of PEK-Inx and PEK-BPFx are shown in Figure 2. All chemical signals correspond correctly to the protons of the polymer by observing the chemical shifts of the peaks and the integrated area ratio. The signal peak at 3.42–2.08 ppm is assigned to the proton of the methylene groups, indicating the successful introduction of the two difluoro-monomers containing methylene into the polymer. In addition, the signal peak at 8.10–6.80 ppm is assigned to the proton on the aromatic rings of the molecular chains. The results show that the obtained polymers are consistent with the designed structures. Furthermore, the chemical structures of PEK-Inx and PEK-BPFx were further confirmed using FT-IR, as shown in Figure 3. The characteristic absorption peak at 2920 cm^−1^ is attributed to the methylene groups (-CH_2_-) of the difluoro-monomer. The characteristic absorption peak appears at 1678 cm^−1,^ corresponding to the stretching vibrational peak of carbonyl groups (C=O). Additionally, the characteristic absorption peak of the ether bond (Ar-O-Ar) appeared at 1230 cm^−1^, and the characteristic absorption peak of the hydroxyl group disappeared. Thus, it was proved that the nucleophilic polycondensation reaction between the bisphenol monomer and the difluoro-monomer was successfully completed. The microscopic structure of PEK-Inx and PEK-BPFx was investigated using WAXD; the results are shown in Figure 4. Both PEK-Inx and PEK-BFPx have typical broad, amorphous diffraction peaks with peak positions in the range of 7.5–30°, demonstrating that all polymers are amorphous. This is mainly attributed to the rigid and twisted side groups in the bisphenol monomer, which prevent the regularity of the polymer molecular chain. In summary, the target polymers were successfully synthesized according to ^1^H NMR, FT-IR, and WAXD characterization.

The molecular weight of PEK-Inx and PEK-BPFx were measured using GPC with an RI monitor. The results of molecular weight and [*η*] of PEK-Inx and PEK-BPFx are listed in Table 1. The number-average molecular (Mn) of PEK-Inx and PEK-BPFx were in the range from 30.2 to 38.3 kDa, the weight-average molecular (Mw) was in the range from 53.1 to 113.2 kDa, the polydispersity index (PDI) were lower than 2.96. In the Ubbelohde viscometer results, the [*η*] was in the range of 0.31–0.45 dL/g, indicating that high molecular weight polymers were synthesized.

### 3.2. Thermal Properties

**Figure 5:**
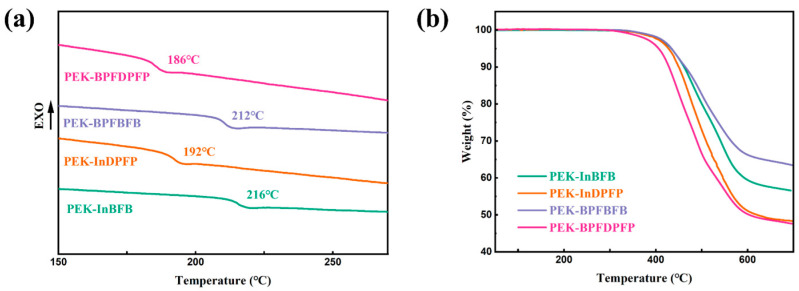
(**a**) DSC curves of PEK-Inx and PEK-BPFx and (**b**) TGA curves of PEK-Inx and PEK-BPFx.

Thermal properties of PEK-Inx and PEK-BPFx were investigated using TGA and DSC in the N_2_ atmosphere. The DSC curve is shown in Figure 5a, and the relevant data are displayed in Table 2. The *T_g_* of PEK-InBFB, PEK-InDPFP, PEK-BPFBFB, and PEK-BPFDPFP were 216, 192, 212, and 186 °C, respectively; the PEK-Inx and PEK-BPFx present excellent thermal resistance. The *T_g_* of PEK-Inx and PEK-BPFx decreases with the increment of the number of methylene groups. This is mainly because the increase in the number of methylene groups improves the flexibility of the polymer and makes the movement of the polymer molecular chains easier. Further, it can be found that all the heat flow curve contains a glass transition process without any melting peaks, indicating that all polymers are amorphous, which is also consistent with the WAXD results.

The TGA curve is shown in Figure 5b, while the date is displayed in Table 2. The PEK-Inx and PEK-BPFx exhibit good thermal stability; the 5% thermal decomposition temperature (*T_d_*_5%_) is in the range of 406–434 °C and the 10% thermal decomposition temperature (*T_d_*_10%_) is in the range of 429–462 °C. It is clearly observed that the PEK-Inx process has better thermal stability than PEK-BPFx. This indicated that the isoindolinone group is a more rigid prudent group compared to the fluorene group. The *T_d_* of resins weakens along with the increment of methylene group contents, which is due to the rigidity of the polymer weakening. Furthermore, the carbon residue rate at 700 °C (C_y700 °C_) of PEK-Inx and PEK-BPFx is above 48%, and the C_y700 °C_ shows the same change trend as *T_d_* with the number of methylene groups elevates. This reason is that the ascended content of methylene groups in the polymer reduces the proportion of aromatic structures. As mentioned above, the PEK-Inx and PEK-BPFx have excellent thermal resistance and thermal stability, which have potential applications.

### 3.3. Mechanical Properties

**Figure 6:**
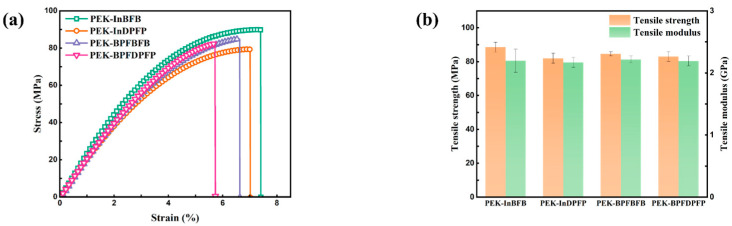
(**a**) The stress–strain curves of PEK-Inx and PEK-BPFx and (**b**) the tensile strength and tensile modulus of PEK-Inx and PEK-BPFx.

The mechanical properties of PEK-Inx and PEK-BPFx films were measured at room temperature. The stress–strain curves are shown in Figure 6a, and the detailed result is listed in Table 3. The tensile strength of PEK-Inx and PEK-BPFx is between 82.0 MPa and 88.5 MPa, the Young’s modulus from 2.17 to 2.21 GPa, and the elongation at the break from 5.54 to 7.94%, respectively. The test results illustrate that the obtained films possess excellent mechanical properties. In addition, the tensile strength and Young’s modulus decrease with the rise of the methylene groups amount due to the diminished stiffness of molecular chains. In conclusion, the four types of poly (aryl ether ketones) films with excellent mechanical properties are obtained.

### 3.4. Solubility Properties

The solubility of PEK-Inx and PEK-BPFx was tested by dissolving 0.01 g of polymer in 1 mL of different solvents at room temperature for 12 h, and the results are presented in Table 4. The PEK-Inx and PEK-BPFx perform favorable solubility; they are dissolved in DMAc, DMF, NMP, CH_2_Cl_2_, and CHCl_3_, except in EA. The reason is that the twisted and rigid isoindolinone and fluorene side groups disrupt the regularity of the molecular chain, increasing the free volume of the polymer and allowing solvent molecules to penetrate more easily into the molecular chain. Furthermore, this favorable solubility gives the PEK-Inx and PEK-BPFx excellent film-forming properties.

### 3.5. Dielectric Properties

**Figure 7:**
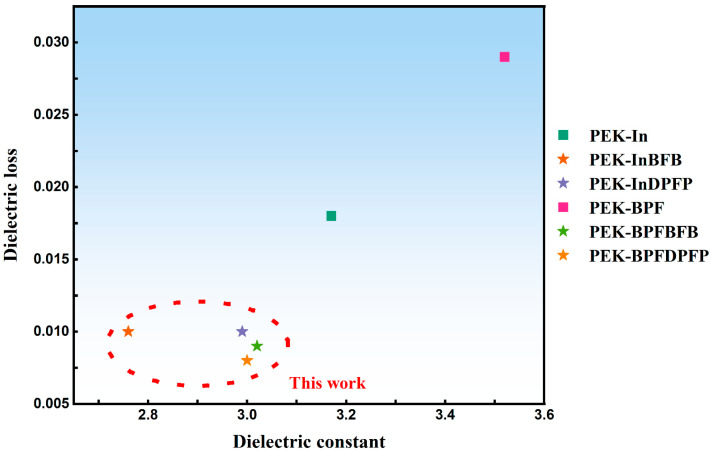
Comparison of dielectric constant and dielectric loss with PEK-In, PEK-Inx, PEK-BPF, and PEK-BPFx at 10 GHz.

The dielectric properties of PEK-Inx and PEK-BPFx were investigated at 10 GHz, and the results are shown in Table 5. The dielectric constant of PEK-Inx and PEK-BPFx films is 2.76 to 3.02, and dielectric loss is 0.009 to 0.010, respectively. The obtained films show a low dielectric constant. This is because the dielectric constant is a parameter that reflects the polarization strength of the dielectric to the polarization response of an external electric field. The introduction of low-polarizability methylene groups effectively decreases the electronic and atomic polarization strengths. Hence, the dielectric constant is lower [30]. Meanwhile, we compared the dielectric properties of PEK-Inx and PEK-BPFx with the reference resins PEK-In and PEK-BPF (the PEK-In and PEK-BPF structures are seen in Figure S5), as shown in Figure 7. It can be clearly observed that both the dielectric constant and dielectric loss of PEK-Inx and PEK-BPFx are lower than those of PEK-In and PEK-BPF, indicating that the introduction of methylene groups into polymers can effectively reduce the dielectric constant and dielectric loss. Presumably, the methylene group contains low polarizability sigma bonds, which further decrease the polarizability of the polymers. Thus, PEK-Inx and PEK-BPFx display lower dielectric constant and dielectric loss compared to PEK-In and PEK-BPF, which do not contain methylene groups. In PEK-BPFx, the dielectric constant reduces with the increase in the number of methylene groups, but in PEK-Inx, the results are reversed. To explain this phenomenon, we constructed the amorphous accumulation cell model of PEK-Inx and PEK-BPFx.

**Figure 8:**
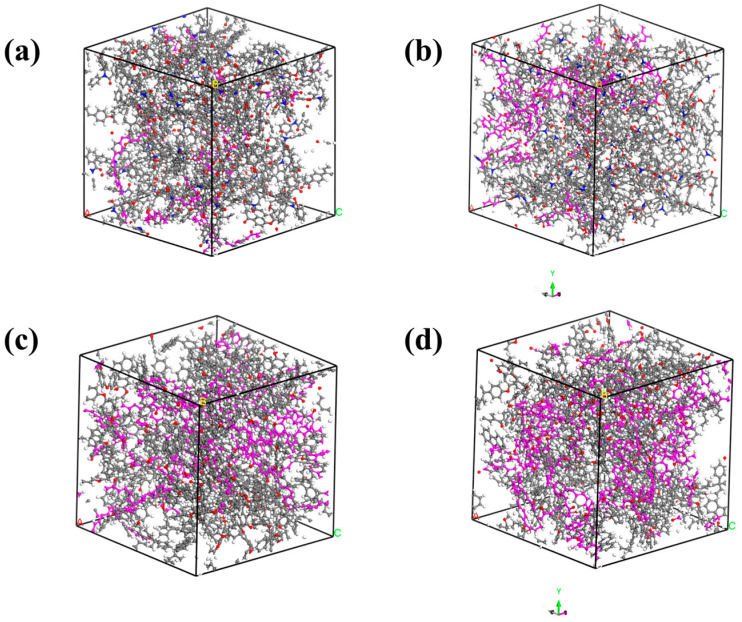
The accumulating models of PEK-Inx and PEK-BPFx, (**a**) PEK-InBFB, (**b**) PEK-InDPFP, (**c**) PEK-BPFBFB, and (**d**) PEK-BPFDPFP.

The amorphous accumulating cell models of PEK-Inx and PEK-BPFx were constructed using Materials Studio software referring to the previous literature [31,32,33]. The results are listed in Table 6. and the corresponding models are shown in Figure 8. It is found that the fractional free volume (FFV) of PEK-Inx and PEK-BPFx declines with the increment of the number of methylene groups. It is obvious to explain the relationship between the dielectric constant and the number of methylene groups by simulating the FFV. There are two factors that affect their dielectric constant for polymers. The first factor is the polarizability of the polymer, and lowering its polarizability would reduce the dielectric constant. Another factor is the FFV of the polymer, and enhancing its FFV can reduce the dielectric constant. In PEK-Inx, although the polarizability of the polymer declined along with the elevating number of methylene groups, at the same time, the FFV of the polymer decreased. The change in polymer FFV had a stronger effect on the dielectric constant. Therefore, the dielectric constant of the polymer did not reduce with the increasing number of methylene groups. On the contrary, in PEK-BPFx, it might be due to the more powerful effect of the change in polymer polarizability on the dielectric constant, which leads to a different change trend for PEK-Inx and PEK-BPFx. In summary, the PAEK that we designed with different numbers of methylene groups had high potential utility for microelectronic devices.

## 4. Conclusions

In this work, novel PAEK-containing methylene groups (PEK-Inx, PEK-BPFx) were synthesized via S_N_2 nucleophilic polycondensation reaction. The results show that the resultant polymers have excellent thermal resistance, mechanical, and solubility properties. The low polarizability methylene group allows the polymer to exhibit low dielectric constant and dielectric loss at 10 GHz, making it possible to become a candidate for low dielectric materials. Additionally, we depict the relationship between the dielectric constant and the amount of methylene groups by molecular dynamics simulation. In conclusion, it is an effective method to reduce the dielectric constant and dielectric loss of polymers by introducing low polarizability methylene groups into the molecular chain.

## Figures and Tables

**Table 1:** Molecular weights and intrinsic viscosity [*η*] of the PEK-Inx and PEK-BPFx.

Polymers	Monomers	Mn ^a^ (kDa)	Mw ^a^ (kDa)	PDI ^a^	[*η*] ^b^ (dL/g)
PEK-InBFB	PPPBP + BFB	30.2	53.1	1.76	0.45
PEK-InDPFP	PPPBP + DPFP	37.3	98.2	2.63	0.44
PEK-BPFBFB	BPF + BFB	32.6	69.2	2.13	0.38
PEK-BPFDPFP	BPF + DPFP	38.3	113.2	2.96	0.31

^a^: The molecular weights measured in THF solution. ^b^: The intrinsic viscosity measured in DMF solution (0.5 g/dL) at 25 °C.

**Table 2:** Thermal performance of the PEK-Inx and PEK-BPFx.

Polymers	*T_d_*_5%_ (°C)	*T_d_*_10%_ (°C)	C_y700 °C_ (%)	*T_g_* (°C)
PEK-InBFB	434	462	57	216
PEK-InDPFP	418	441	48	192
PEK-BPFBFB	434	464	63	212
PEK-BPFDPFP	406	429	48	186

**Table 3:** Mechanical performance of the PEK-Inx and PEK-BPFx.

Polymers	Young’s Modulus (GPa)	Tensile Strength (MPa)	Elongation at Break (%)
PEK-InBFB	2.20 ± 0.18	88.5 ± 2.9	7.94 ± 1.19
PEK-InDPFP	2.17 ± 0.08	82.0 ± 3.0	7.55 ± 0.56
PEK-BPFBFB	2.21 ± 0.06	84.6 ± 1.2	6.79 ± 0.47
PEK-BPFDPFP	2.19 ± 0.08	83.1 ± 2.9	5.54 ± 0.17

**Table 4:** Solubility of the PEK-Inx and PEK-BPFx.

Polymers	Solubility ^a^					
	NMP	DMAc	DMF	CH_2_Cl_2_	CHCl_3_	EA
PEK-InBFB	+	+	+	+	+	-
PEK-InDPFP	+	+	+	+	+	-
PEK-BPFBFB	+	+	+	+	+	-
PEK-BPFDPFP	+	+	+	+	+	-

^a^: solubility test was performed at 0.01g/mL at room temperature. + soluble at room temperature - Insoluble.

**Table 5:** Dielectric constant and dielectric loss of the PEK–Inx and PEK-BPFx.

Polymers	10 GHz	
	Dielectric Constant	Dielectric Loss
PEK-In	3.17	0.018
PEK-InBFB	2.76	0.010
PEK-InDPFP	2.99	0.010
PEK-BPF [28]	3.52	0.029
PEK-BPFBFB	3.02	0.009
PEK-BPFDPFP	3.00	0.008

**Table 6:** The parameters and results of accumulating model simulation.

Polymers	Density (g cm^−3^)	Occupied Volume (Å3)	Free Volume (Å3)	FFV (%)
PEK-InBFB	1.3	75,998.57	2493.76	3.18
PEK-InDPFP	1.3	78,480.52	1855.04	2.30
PEK-BPFBFB	1.3	70,892.95	4236.54	5.64
PEK-BPFDPFP	1.3	72,888.09	4033.12	5.24

## Data Availability

Data is available on request from the corresponding author.

## Supplementary Materials

The following supporting information can be downloaded at: https://www.mdpi.com/article/10.3390/polym15214330/s1. Figure S1: ^1^H NMR spectrum of BFB. Figure S2: High-resolution gas chromatography–mass spectroscopy of BFB. Figure S3: ^1^H NMR spectrum of DPFP. Figure S4: High-resolution gas chromatography–mass spectroscopy of DPFP. Figure S5: Structural formulas for PEK-In and PEK-BPF.
