# Supplementary material for: Amorphous Poly (Aryl Ether Ketones) Containing Methylene Groups with Excellent Thermal Resistance, Dielectric Properties and Mechanical Performance
Source: Polymers (Basel). 2023 Nov 6;15(21):4330. doi: 10.3390/polym15214330 (PMC10650800; doi:10.3390/polym15214330)
Supplement: Supplementary file 1 [file polymers-15-04330-s001.zip › polymers-2616159-supplementary.pdf]

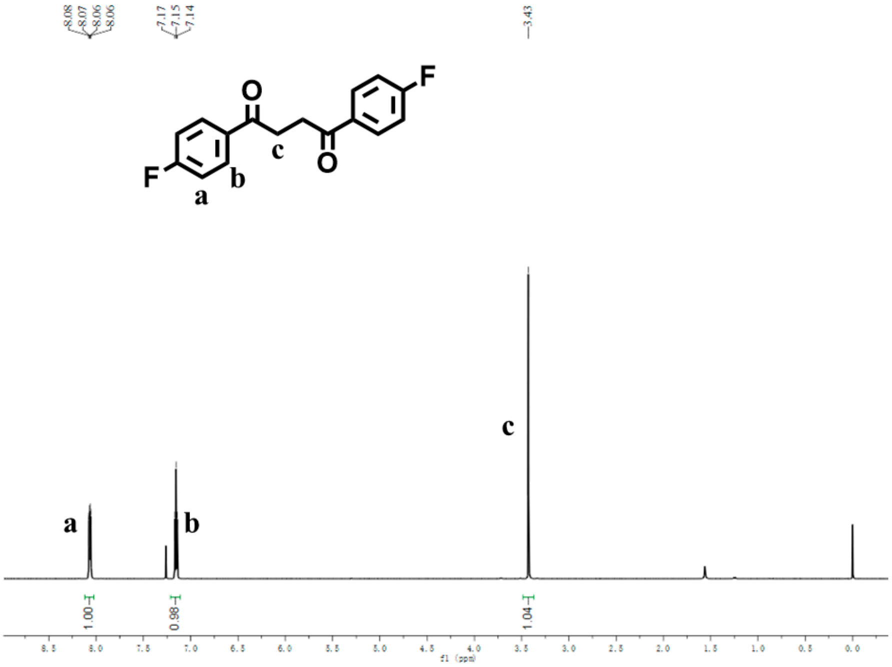

Figure S1. ^{1}H NMR spectrum of BFB.

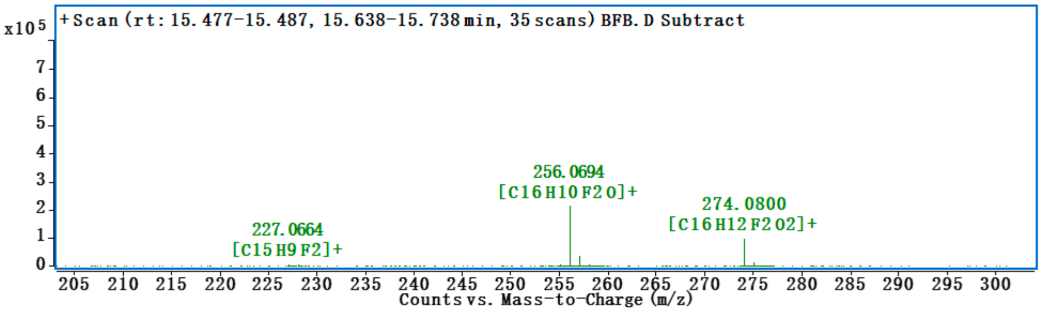

Figure S2. High-resolution gas chromatography–mass spectroscopy of BFB.

Figure. S3. ^{1}H NMR spectrum of DPFP.

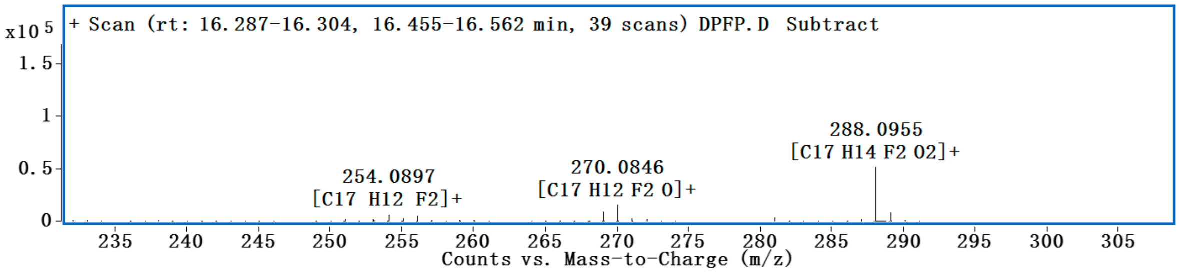

Figure S4. High-resolution gas chromatography–mass spectroscopy of DPFP.

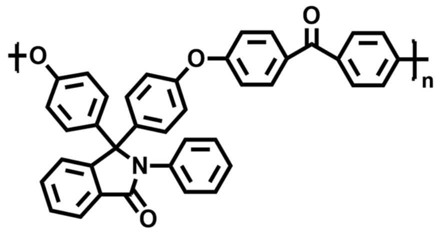

PEK-In

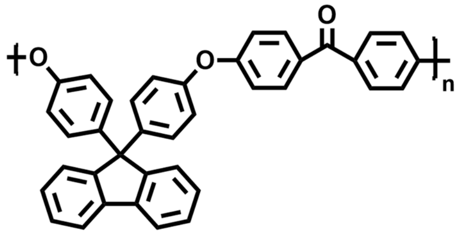

PEK-BPF

Figure S5. Structural formulas for PEK-In and PEK-BPF.
